# Impact of Degree Heterogeneity on Attack Vulnerability of Interdependent Networks

**DOI:** 10.1038/srep32983

**Published:** 2016-09-09

**Authors:** Shiwen Sun, Yafang Wu, Yilin Ma, Li Wang, Zhongke Gao, Chengyi Xia

**Affiliations:** 1 Tianjin Key Laboratory of Intelligence Computing and Novel Software Technology, Tianjin University of Technology, Tianjin, 300384, China; 2 Key Laboratory of Computer Vision and System (Tianjin University of Technology), Ministry of Education, Tianjin, 300384, China; 3 School of Electrical Engineering and Automation, Tianjin University, Tianjin, 300072, China

## Abstract

The study of interdependent networks has become a new research focus in recent years. We focus on one fundamental property of interdependent networks: vulnerability. Previous studies mainly focused on the impact of topological properties upon interdependent networks under random attacks, the effect of degree heterogeneity on structural vulnerability of interdependent networks under intentional attacks, however, is still unexplored. In order to deeply understand the role of degree distribution and in particular degree heterogeneity, we construct an interdependent system model which consists of two networks whose extent of degree heterogeneity can be controlled simultaneously by a tuning parameter. Meanwhile, a new quantity, which can better measure the performance of interdependent networks after attack, is proposed. Numerical simulation results demonstrate that degree heterogeneity can significantly increase the vulnerability of both single and interdependent networks. Moreover, it is found that interdependent links between two networks make the entire system much more fragile to attacks. Enhancing coupling strength between networks can greatly increase the fragility of both networks against targeted attacks, which is most evident under the case of max-max assortative coupling. Current results can help to deepen the understanding of structural complexity of complex real-world systems.

Complex network is an important tool used to describe and analyze the structure and dynamical behaviors of complex systems^1^,^2^,^3^. Since real-world complex systems are becoming increasingly dependent on one another, the study of interdependent networks has become another new active topic in network science^4^,^5^. Modern critical infrastructures are representative examples of interdependent systems, such as water supply, power stations, fuel supply, transportation, communication, etc. For example, considering the interdependence between power grids and communication networks, power grids need communication networks to transmit control signals and communication networks need power grids to provide power supply. The investigation of interdependent networks has led to new discoveries that cannot be explained using a single-network framework^6^,^7^,^8^,^9^,^10^,^11^,^12^,^13^,^14^.

We focus on one fundamental property of interdependent networks: attack vulnerability. Albert *et al*.^15^ raised the study of complex networks under attacks, they found the “*robust yet fragile*” generic property of scale-free networks: scale-free networks display an unexpected degree of robustness to random failures, however, these networks are extremely vulnerable to intentional attacks. Their research has triggered numerous theoretical and experimental works in this topic^16^,^17^,^18^,^19^,^20^,^21^,^22^,^23^,^24^,^25^. However, most previous studies mainly focused on single, isolated networks. Based on percolation theory, recently, Buldyrev *et al*. proposed a general framework to investigate the attack resilience of a system composed of two networks whose nodes are mutually dependent^26^,^27^. In this model, attack on nodes is simulated by random node removal from one network. Due to the existence of interdependent links, an initial failure of only a small fraction of nodes in one network can lead to an iterative cascade of failures that cause both networks to become fragmented. Moreover, interdependent systems can react to random failures in a manner that is totally different from single networks, i.e., an interdependent system can exhibit a first-order (discontinuous) phase transition instead of the second-order (continuous) phase transition which is typical for single networks^16^,^18^.

In reality, the topological features should be taken into account for a complete description of the networks. Thus it is of great importance to explore the effects of the structural properties of networks on attack vulnerability of interdependent systems. Several recent papers focused on the influence of clustering^28^,^29^,^30^,^31^. Huang *et al*.^28^ established a fully interdependent system of two networks with tunable clustering and found that clustering significantly increases the vulnerability. The impact of clustering on partially interdependent systems is also investigated^29^ and the percolation behaviors of clustered networks with partial support-dependence relations are analyzed based on the percolation theory^30^. Clustering coefficient is found to have a significant impact on robustness of the system particularly with strong coupling strength, however weak coupling strength can induce little influence^31^. The degree distribution is one of the most fundamental and important properties of complex networks. For example, single network with a broader degree distribution can be more robust to random failures, however, for interdependent networks, the broader the distribution is, the more vulnerable the networks become to random failures^32^,^33^. Zhou *et al*.^34^ found that the internal node correlations in each of the two interdependent networks significantly changes the critical density of failures that triggers the total disruption of the two-network system. In particular, the assortativity, i.e., the likelihood of nodes with similar degree to be connected within a single network, decreases the robustness of the entire system^35^,^36^. Additionally, Yuan *et al*.^37^ study the effect of the breadth of the degree distributions on network robustness by comparing two different attacking strategies: localized attack and random attack.

In this study, we continually focus on the effect of degree distribution on attack vulnerability of single and interdependent networks. An typical evolving network model, named extended Barabási-Albert model (*eBA*)^38^, is employed as network component of an interdependent system. *eBA* model is one of the variants of *BA* model^39^ with a parameter *p*(*p* ∈ [0, 1]). By varying *p*, the heterogeneity of degree distributions of corresponding networks can be controlled. Considering that in real-world coupled systems not every node in one network depends on another network, thus a parameter named coupling strength *q*, defined here as the fraction of network nodes that are dependent on the other network, is introduced in the interdependent network model. Furthermore, other than random interdependency between networks, coupling preference is also taken into consideration on the performance of interdependent systems. Additionally, previous studies mainly focused on the impact of degree distribution on interdependent networks under random attacks, while we extend the study to the case of the more realistic attacking strategy, targeted attack on high-degree nodes.

## Results

### Vulnerability of single networks

**Figure 1:**
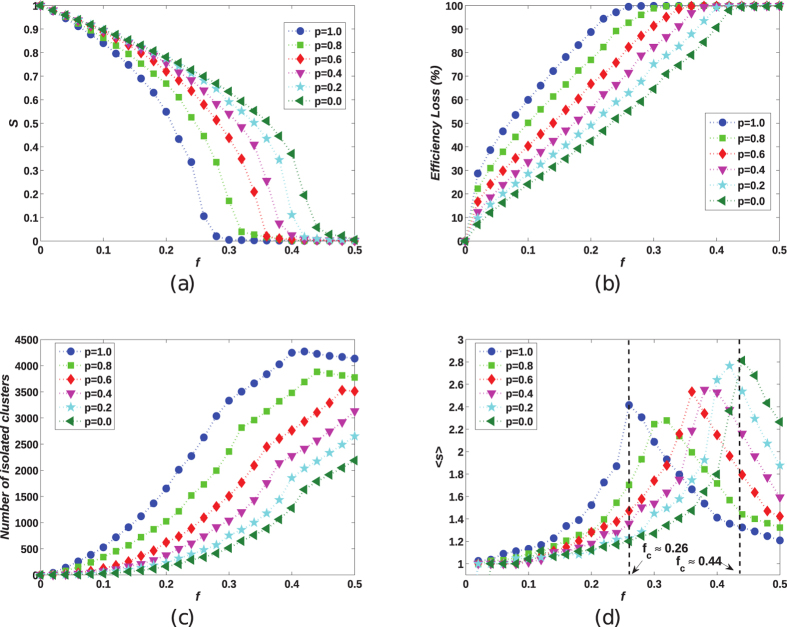
Vulnerability of single *eba* networks with different *p* after a fraction *f* of nodes removed from the networks. (**a**) The relative size *S* of the giant connected component; (**b**) Efficiency loss (*el*); (**c**) Number of isolated connected components (*Ns*); (**d**) Average size of isolated connected components (〈*s*〉). All the networks are with *N* = 10000 and 〈*k*〉 = 6. Each point is averaged over 10 independent realizations.

Firstly, numerical simulations are performed to investigate the effect of degree heterogeneity on single complex networks. The responses of single *eBA* networks under targeted node removal are exhibited in Fig. 1. All the initial networks (*N* = 10,000 and 〈*k*〉 = 6) are constructed by *eBA* model with *m* = 3. Each point is averaged over 10 independent realizations.

The relative size *S* of the giant connected component is usually used to probe the functional integrity of networks after attack. *S* is defined to be *S* = *N*′/*N* where *N*′ and *N* denotes the number of nodes in the largest connected component and that in the initial network, respectively. Obviously, the larger *S* is, the more nodes remain in the largest component, which indicates the system is more robust under attacks. Figure 1(a) shows the relative size *S* of the giant connected components of single *eBA* networks with different parameter *p* after a fraction *f* of nodes removed from the networks. *S* decreases from *S* = 1 as *f* increases. At critical point *f* = *f*_*c*_, *S* ≈ 0, indicating that the network breaks into tiny isolated clusters. It can be observed from Fig. 1(a) that *eBA* networks with higher values of *p* are more vulnerable resisting targeted attack on high-degree nodes.

Also, considering the efficiency loss caused by the removal of nodes and links, efficiency loss *el* of the residual network after attack monotonically increases with *f* (Fig. 1(b)). Different changes of *el* with *f* can demonstrate the transitional behaviors of the vulnerability of *eBA* networks with different *p* against attacks. As observing from Fig. 1(b), removing the same percent of high-degree nodes from the networks will bring more efficiency loss on networks with higher values of *p*. The results demonstrate that targeted attack can bring more damage to the networks which are more heterogenous in connectivity.

In order to describe the fragment process of networks after attack in more detail, *Ns*, the number of isolated components breaking off from the main body, and 〈*s*〉, the average size of these isolated components, can be examined. For *eBA* networks with different parameter *p*, Fig. 1(c) shows the the changes of *Ns* as functions of *f*. As more nodes are removed from the networks, more and more nodes breaking off from the giant connected components, thus, *Ns* increases with *f*. It can be observed that with the same value of *f, Ns* of networks with higher values of *p* are larger. For example, when *f* = 0.25, *Ns* ≈ 250 for *eBA* network with *p* = 0.0, while *Ns* increases with *p*, and for network with *p* = 1.0, *Ns* is increased to *Ns* ≈ 2600.

Meanwhile, as shown in Fig. 1(d), there exits a critical threshold value *f*_*c*_ at which 〈*s*〉 reaches its maximum value and the phase transition occurs according to the percolation theory^2^,^16^,^17^,^18^. During network fragmentation process, for small *f*, single nodes break off from the main body, so 〈*s*〉 ≈ 1. With the increase of *f*, the size of the fragments that fall off the main body increases, thus 〈*s*〉 increases. At *f* = *f*_*c*_, the giant component breaks into small pieces quickly *S* ≈ 0, and the size of fragments 〈*s*〉 peaks. As the continue removal of nodes *f* > *f*_*c*_, isolated components breaks apart continually resulting to a decreasing 〈*s*〉. As shown in Fig. 1(d), as *p* is increased, *f*_*c*_ becomes smaller, which indicates that corresponding network become more fragile. For example, when *p* = 0.0, *f*_*c*_ ≈ 0.44, however, *f*_*c*_ is observed to be about 0.26 when *p* = 1.0. Moreover, 〈*s*〉 increases more drastically with increasing *p*.

To conclude, the numerical results in Fig. 1 show that, under intentional attacks, the fragility of the *eBA* networks also show a transition between that of the scale-free network (*p* = 1.0) and of the exponential network (*p* = 0.0). Moreover, heterogenous networks, that is, networks with higher values of *p*, are found to be more fragile resisting targeted attack on high-degree nodes.

### Vulnerability of fully interdependent networks

Next, we explore the influence of both degree heterogeneity and interdependency on the fully interdependent *eBA* networks, which corresponds to the case of coupling strength *q* = 1.0. According to the interdependent system model mentioned above, an interdependent system are constructed firstly in the numerical simulation. Two different networks, network *A* and network *B*, are separately constructed according to *eBA* model with parameters *m* = 3 and *N* = 2000. Meanwhile, the degree heterogeneity of each network can be controlled by parameter *p*, as *p* is changed from 0 to 1, the extent of degree heterogeneity of each network is enhanced greatly.

Additionally, different types of interdependency links are taken into account in the construction of interdependent networks. As for random coupling, after the construction of two networks *A* and *B*, randomly choose a node in network *A* and a node in network *B* and set up an interdependent link between them, repeat this process until *N* interdependent links are added. Meanwhile, two kinds of coupling preference are also investigated. The first one, which is referred to as assortative coupling, means sorting nodes in network *A* and *B* in the descending order of node degree and connecting the nodes in *A* and *B* one by one. The other one, referred to as *disassortative coupling*, means sorting nodes in network *A*(*B*) in the descending(ascending) order of node degree and connecting them one by one.

**Figure 2:**
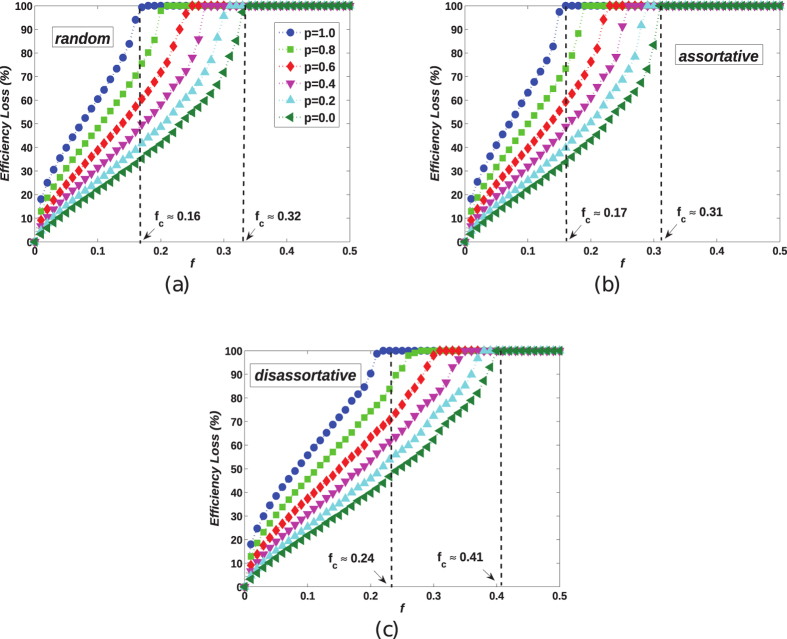
Vulnerability of interdependent *eba* networks with different *p* after a fraction *f* of nodes removed from the networks with coupling strength *q* = 1.0. Different coupling types are considered separately: (**a**) random coupling; (**b**) assortative coupling; (**c**) disassortative coupling. All the networks are with *N* = 2000 and 〈*k*〉 = 6. Each point is averaged over 10 independent realizations. The legends in (**b**,**c**) are the same as those of (**a**).

Figure 2 show the responses of fully interdependent *eBA* networks under targeted attacks on high-degree nodes with random (Fig. 2(a)), assortative (Fig. 2(b)) and disassortative (Fig. 2(c)) coupling, respectively. Considering the impact of degree heterogeneity of interdependent networks, as shown in Fig. 2, with all the three kinds of coupling, efficiency loss *el* of networks increases more rapidly with higher value of *p*, which demonstrates that corresponding networks are more vulnerable to attacks in targeted ways. Note that, this behavior is consistent with that of isolated *eBA* networks (Fig. 1).

Previous study has found that due to the existence of dependency links, a system composed of two interdependent networks is much more fragile than each network in isolation^26^,^27^. For isolated *eBA* networks, curves in Fig. 1(b) show the efficiency losses with increasing *f*. At the critical values *f*_*c*_ the communication efficiency is totally lost (i.e., *el* ≈ 100%) and the whole network collapses. It can be clearly observed that as parameter *p* is changed from 0 to 1, *fc* ∈ [0.26, 0.44]. However, in Fig. 2(a), the responses of interdependent networks are different, that is, *f*_*c*_ ≈ 0.32 when *p* = 0.0 and *f*_*c*_ ≈ 0.17 when *p* = 1.0. The decrease of *f*_*c*_ indicates that dependency links between networks make both networks become more vulnerable with respect to attacks on nodes.

Coupling preference can also greatly affect the properties and behaviours of complex interdependent networks. Rather than random coupling (Fig. 2(a)), attack vulnerabilities of networks with assortative and disassortative coupling are presented in Fig. 2(b,c) respectively. Assortative coupling can bring more efficiency loss caused by the same number of removed nodes compared with disassortative coupling. For example, at *f* = 0.1, for networks with *p* = 1.0, the efficiency loss is 60% in Fig. 2(b), however, for the same network with disassortative coupling (Fig. 2(c)), at *f* = 0.1, the efficiency loss is deceased to 50%. Moreover, compared with disassortative coupling, the values of *f*_*c*_ are observed to be smaller than those of networks with the same parameters of *m, N* and *p* under assortative coupling. For example, when *p* = 0.0, *f*_*c*_ ≈ 0.32 for assortative coupling (see Fig. 2(b)) but *f*_*c*_ ≈ 0.41 under the case of disassortative coupling (see Fig. 2(c)). All the simulation results strongly demonstrate that assortative coupling makes interdependent networks more vulnerable compared with disassortative coupling.

### Vulnerability of partially interdependent networks

As for partially interdependent networks, a parameter *q*(0.0 ≤ *q* ≤ 1.0) is employed to control the coupling strength between two networks. Moreover, different coupling types are considered separately including random, *max-max, min-min, max-min* and *min-max* coupling.

The critical values *f*_*c*_ of removed nodes from the networks, at which the efficiency loss is almost 100%, is used as an important quantity to measure the vulnerability of corresponding systems. Obviously, the smaller the value of *f*_*c*_ is, the more vulnerable the network is, and *vice visa*.

**Figure 3:**
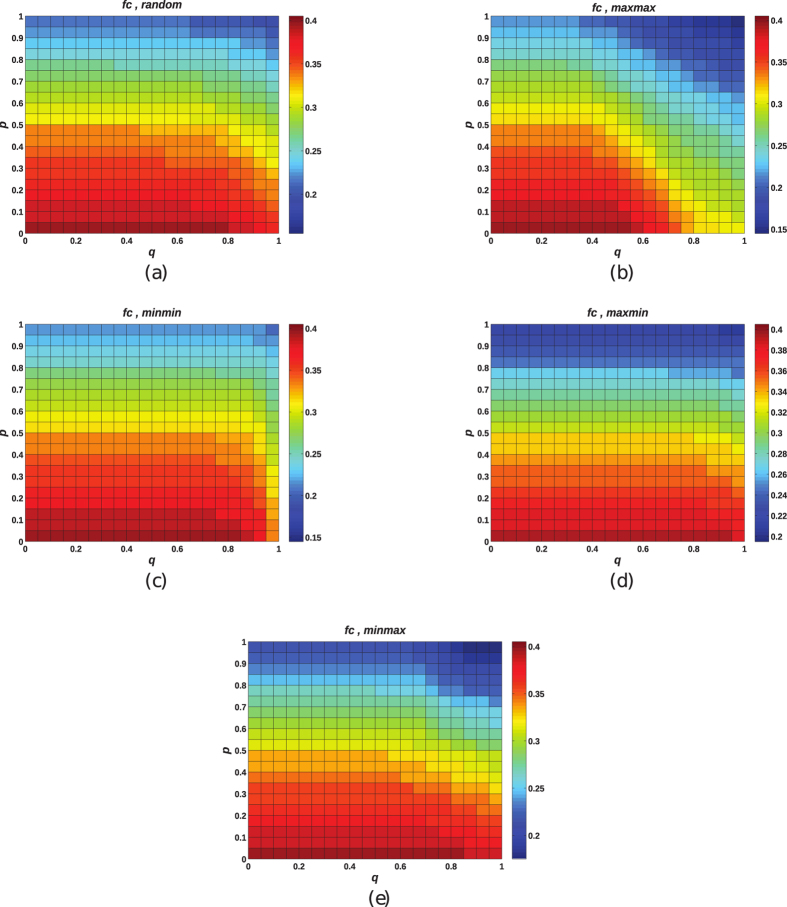
The critical values *f*_*c*_ as a function of the coupling strength *q* and model parameter *p*. Different coupling types are considered separately: (**a**) random coupling; (**b**) *max-max* coupling; (**c**) *min-min* coupling; (**d**) *max-min* coupling; (**e**) *min-max* coupling. All the networks are with *N* = 2000 and 〈*k*〉 = 6. Each point is averaged over 10 independent realizations.

In order to explore the influence of both degree heterogeneity and interdependency, numerical simulations are performed to examine the responses of partially interdependent networks. Figure 3 presents the values of *f*_*c*_ as functions of coupling strength *q* and parameter *p* of interdependent networks with different coupling types: random coupling (Fig. 3(a)), *max-max* coupling (Fig. 3(b)), *min-min* coupling (Fig. 3(c)), *max-min* coupling (Fig. 3(d)), *min-max* coupling (Fig. 3(e)). All the networks are with *N* = 2000 and 〈*k*〉 = 6. Each point is averaged over 10 independent realizations.

From Fig. 3, when *p* is fixed, that is, the two interdependent networks are with the same extent of degree heterogeneity, with the increase of coupling strength *q, f*_*c*_ is observed to decrease. The most evident decrease of *f*_*c*_ can be observed under the case of *max-max* coupling (Fig. 3(b)). Since a smaller value of *f*_*c*_ indicates that corresponding network become more vulnerable against node attacks, the results demonstrate that strong interdependency between networks induces more vulnerability. Thus, due to the existence of dependency links, a system composed of two interdependent networks is much more fragile than each network in isolation no matter what kind of coupling preference is.

Additionally, with fixed *q, f*_*c*_ is observed to decrease as *p* is increased, thus confirming that for partially interdependent networks, when networks become more heterogenous in connectivity, they are also more vulnerable to resist node attacks. The vulnerability is basically rooted in the network’s connectivity. For heterogenous networks (*p* = 1.0), the connectivity is ensured by a few high-degree nodes, whose removal drastically alters the network’s topology and decrease the ability of the remaining nodes to communicate with each other. While in homogeneous network (*p* = 0.0), most nodes have approximately the same number of connections and contribute equally to the integrity of the topology. Due to the absence of nodes with large connections 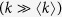, targeted removal of hub nodes does not affect the structure of remaining nodes as drastically as in heterogenous networks.

The most vulnerable case occurs under the case of *p* = 1.0, *q* = 1.0 with *max-max* coupling (see Fig. 3(b)). When targeted attack is initiated in one network, the *max-max* coupling makes node failures quickly propagate between high-degree nodes of each networks, thus leading to rapid collapse of both networks. Nevertheless, the interdependent system with two networks coupled in a *min-min* mode are more robust than *max-max* and *min-max* coupling in particular for strong coupling strength *q* (see Fig. 3(e)). Since with *min-min* coupling low-degree nodes in network *A* and *B* are connected to each other, the failure of low-degree nodes in network *A* can only affect low-degree nodes in network *B*, having little effects on high-degree nodes in *B*. While, in *max-max* and *min-max* coupling, the failures of low-degree nodes in network *A* can lead to the failures of all the high-degree nodes in *B*, thus, making cascading failures propagate more quickly between two networks.

## Discussion

The attack vulnerability of networks can be greatly influenced by their degree distributions and in particular by degree heterogeneity. In order to deeply understand the role of degree heterogeneity upon interdependent networks, An typical evolving network model, named extended Barabási-Albert model, is employed as network component of an interdependent system. *eBA* model can generate networks displaying a transition from exponential to power-law form with respect to degree distributions. Also a parameter named coupling strength *q*, defined as the fraction of network nodes that are dependent on the other network, is introduced in the interdependent network model. Furthermore, other than random interdependency between networks, coupling preference is also taken into consideration on the performance of interdependent systems. In order to better describe the responses of interdependent networks after node removal, a new quantity concerning the communication efficiency is introduced.

Numerical simulation results demonstrate that degree heterogeneity can significantly increase the vulnerability of both single and interdependent networks. Networks with heterogenous degree distribution are more vulnerable against targeted attacks on high-degree nodes, and this result also holds for interdependent networks. Moreover, it is found that interdependent links between two networks make the entire system much more fragile to attacks. Enhancing coupling strength between networks can greatly increase the fragility of both networks against targeted attacks, which is most evident under the case of max-max assortative coupling. These results can improve the deep understanding of structural complexity of complex real-world systems, also give some insight to the guidance of designing resilient infrastructures.

## Methods

### Constructing Extended Barabási-Albert Networks

Network growth and preferential attachment(*PA*) are argued to the emergence of the power-law degree distribution (i.e., 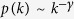) in Barabási-Albert(*BA*) scale-free networks^39^. Extended BA model(*eBA*)^38^ is one of the variants of *BA* model by introducing a parameter *p*(*p* ∈ [0, 1]). By varying *p*, the heterogeneity of degree distributions of corresponding networks can be controlled.

The iterative algorithm of *eBA* model is outlined as follows. Starting from *m*_0_ fully connected nodes, at each step *t*, a new node is added to the network with *m*(*m* ≤ *m*_0_) edges that link to *m* different nodes already existing in the network. The *m* links are attached in two different ways: i) with probability *p*, the *PA* rule is used, that is, the new node is connected to an existing node *i* according to the probability Π_*i*_ = *k*_*i*_/∑_*j*_*k*_*j*_; ii) with probability (1 − *p*), the new node is connected to a randomly chosen node.

**Figure 4:**
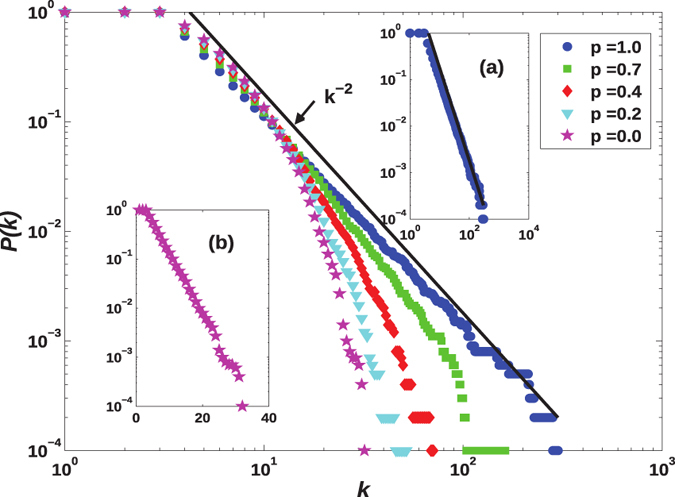
Cumulative degree distribution *P*(*k*) of *eBA* evolving networks with *N* = 10,000 and 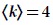 for different parameter *p*. In panel (a) (log-log scale), *P*(*k*) follows a power-law form, which corresponds to one special case of *eBA* networks (*p* = 1.0). Panel (b) (in semi-log scale) presents the other special case of *eBA* networks (*p* = 0.0), whose degree distribution follows a exponential form. A higher value of *p* makes corresponding network more heterogeneous in connectivity.

The cumulative degree distributions, defined as 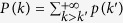, of *eBA* networks are shown in Fig. 4. It can be observed that the probability *p* in *eBA* model has great effect on the network’s degree distributions. Here, two special cases exist. If *p* = 1.0, the model reduces to the standard *BA* network with a degree distribution following a power-law form (see panel (a) in Fig. 4). On the other hand, if *p* = 0.0, the preferential attachment mechanism does not take effect and the model results in a network with a degree distribution following an exponential form: 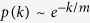 (see panel (b) in Fig. 4). In addition, noticeably, as *p* is changed from 0 to 1, corresponding networks display transitional behaviors from exponential to power-law form with respect to degree distributions.

**Figure 5:**
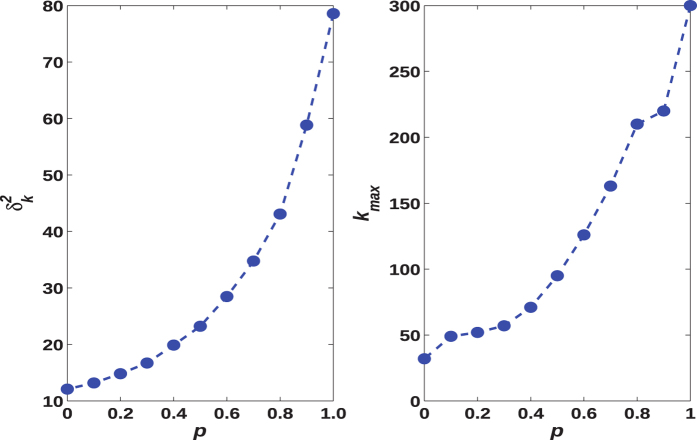
The dependencies of 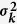 and *k*_*max*_ on parameter *p* of *eBA* networks with *N* = 10,000 and 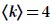.

In order to further study the effects of *p* on degree heterogeneity, two important indicators, 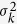 and *k*_*max*_ of the resultant networks are examined. *k*_*max*_ means the maximal value of the node degree in the whole network. 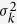 is defined to be the variance of node degree sequence, i.e., 
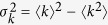
. Figure 5 shows the dependencies of 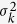 and *k*_*max*_ on *p*. For 0 ≤ *p* ≤ 1, as *p* increases, 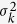 is observed to increase monotonously, implying the increase of degree heterogeneity (see Fig. 5(a)). Meanwhile, with increasing *p, k*_*max*_ also becomes larger (see Fig. 5(b)). The increase of *k*_*max*_ with increasing *p* indicates the emergence of hub nodes, which have much more connections than the others. These results verify that a higher value of *p* makes corresponding *eBA* network more heterogeneous in connectivity.

### Establishing Interdependent Network System

Following the framework established by Buldyrev *et al*.^26^, A partially interdependent system composed of two networks is proposed. Let network *A* and *B* be *eBA* networks with the same size *N*_*A*_ = *N*_*B*_ = *N* and the same average node degree 〈*k*_*A*_〉 = 〈*k*_*B*_〉 = 〈*k*〉. Also, *A* and *B* are with the same parameter *p*, i.e. *p*_*A*_ = *p*_*B*_ = *p*. Apparently, as *p* is changed from 0 to 1, the extent of heterogeneity of degree distributions of each network is changed greatly, i.e. a higher *p* makes corresponding network become more heterogeneous concerning degree distribution. Note that only one-to-one and symmetric interdependency is considered, which means that node *a*_*i*_ in network *A* only depends on one node *b*_*j*_ in *B* and vice visa. For partially coupling, only a fraction *q* of nodes in network *A* and *B* depends on each other. 0 ≤ *q* ≤ 1 and *q* = 1 corresponds to the case of fully coupling.

**Figure 6:**
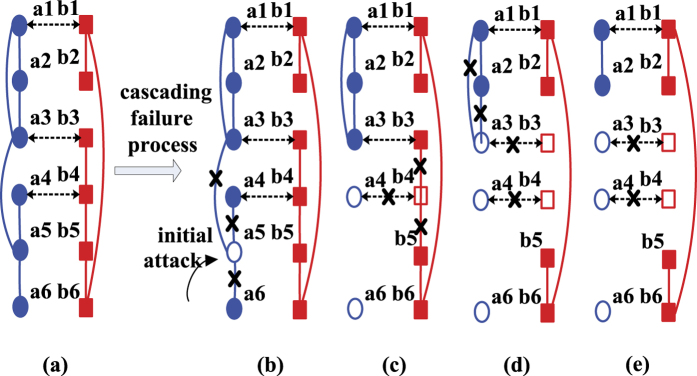
Illustration of an interdependent system composed of two networks and the cascading failure process caused by node removal from one network. The initial system is shown in (**a**). Nodes in network *A* are represented by blue circles ({*a*_*i*_|1 ≤ *i* ≤ 6}) and nodes in network *B* are represented by red squares ({*b*_*j*_|1 ≤ *j* ≤ 6}). The intra-links in each network are represented as solid lines and the interdependent links between networks are represented as dashed lines. (**b**–**e**) Illustrate the iterative process of a cascade of failures induced by an initial attack on a single node *a*_5_ in network *A*.

A simple example of an interdependent system consisting of two networks *A* and *B* is shown in Fig. 6(a). Nodes in network *A* are represented by blue circles ({*a*_*i*_|1 ≤ *i* ≤ 6}) and nodes in network *B* are represented by red squares ({*b*_*j*_|1 ≤ *j* ≤ 6}). The intra-links in each network are represented as solid lines and the interdependent links between networks are represented as dashed lines. Figure 6(b–e) illustrate the iterative process of a cascade of failures induced by an initial attack on a single node *a*_5_ in network *A*. When *a*_5_ fails, all the intra-links (*a*_3_-*a*_5_),(*a*_4_-*a*_5_) and (*a*_6_-*a*_5_) in network *A* fail (Fig. 6(b)). This disconnects nodes *a*_4_ and *a*_6_ from the largest connected component of network *A* and therefore *a*_4_ and *a*_6_ fail. Due to the interdependency between nodes *a*_4_ and *b*_4_, the failure of *a*_4_ triggers the failures of *b*_4_ and all its direct links (*b*_3_-*b*_4_) and (*b*_4_-*b*_5_) (Fig. 6(c)), which makes node *b*_3_ disconnected from the largest connected component of network *B*, hence *b*_3_ fails. The failure of *b*_3_ leads to the failures of the interdependent link (*a*_3_-*b*_3_), node *a*_3_ and two links (*a*_1_-*a*_3_), (*a*_2_-*a*_3_) (Fig. 6(d)). This procedure will not stop until no further node elimination occurs. The system eventually stabilises with the largest connected component (*a*_1_, *a*_2_) in network *A* and (*b*_1_, *b*_2_, *b*_5_, *b*_6_) in network *B* (Fig. 6(e)).

Furthermore, other than random interdependency between networks, coupling preference is also taken into consideration in our study:

- Random coupling. Randomly choose a node in network *A* and a node in network *B* and set up an interdependent link between them, Repeat this process until N*q interdependent links are added.
- Assortative coupling. Two different kinds of assortative coupling, referred to as *max-max* and *min-min* coupling, respectively, are considered. Sort nodes in network *A* and *B* in the descending order of node degree, as for *max-max* coupling, connect the fraction *q* of nodes with the highest degree in *A* and the fraction *q* of nodes with the highest degree in *B*; while for *min-min* coupling, the fraction *q* of nodes with the lowest degree in *A* and the fraction *q* of nodes with the lowest degree in *B* are connected.
- Disassortative coupling. Also, Two different kinds of disassortative coupling, *max-min* (the fraction *q* of nodes with the highest degree in *A* connect the fraction *q* of nodes with the lowest degree in *B*) and *min-max* (the fraction *q* of nodes with the lowest degree in *A* connect the fraction *q* of nodes with the highest degree in *B*), are considered.

### A New Vulnerability Measure - *efficiency loss*(*el*)

When nodes are gradually damaged due to random failures or targeted attacks, a network may be split into several unconnected components. Thus, the vulnerability of networks is mainly measured by the connectivity integrity of the networks. Several measures are commonly used including the relative size *S* of the giant connected component, the number of isolated connected components *Ns*, the average size 〈*s*〉 of connected components except the largest one, and the critical fraction *f*_*c*_ of nodes attacked at which the whole network collapses completely.

However, in realistic cases, these measures may overlooks situations in which the networks suffer from a big damage but they are not completely collapsing. Moreover, other than the study on the connectivity integrity, other properties of the residual nodes and links after attack should also be explored. Thus, in our study, a new quantity, aiming at measuring the communication efficiency of the residual network after attack, is introduced, which is used as an important vulnerability measure of interdependent networks.

Communication efficiency is one of the important quantities to measure how efficiently the information is exchanged over the whole network^21^,^40^. Suppose that information is exchanged between every pair of nodes and transmitted along the shortest path connecting them, communication efficiency *ε*_*ij*_ is assumed to be inversely proportional to the shortest distance: *ε*_*ij*_ = 1/*l*_*ij*_, here, *l*_*ij*_ denotes the length of shortest path between nodes *i* and *j*. Thus, global communication efficiency *ε* of network *G* is defined as the average of *ε*_*ij*_ over all pair of nodes, i.e., *ε* = ∑_*i*≠*j*_*ε*_*ij*_ /(*N*(*N* − 1)), where *N* is the total number of nodes in the network.

Once *ε*(*G*) is defined as a measure of performance of network *G*, the damage caused by the removal of some components(node and/or edges) can be naturally evaluated by the a new measure, *efficiency loss*(*el*), which is defined as


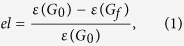


where *ε*(*G*_0_) is the efficiency of the initial network before any attack and *ε*(*G*_*f*_) is the final efficiency that is reached by the network due to the breakdown. Apparently, under the same level of damage, a larger value of *el* means that corresponding network is more vulnerable resisting attacks.

## Additional Information

**How to cite this article**: Sun, S. *et al*. Impact of Degree Heterogeneity on Attack Vulnerability of Interdependent Networks. *Sci. Rep.* **6**, 32983; doi: 10.1038/srep32983 (2016).
